# Effect of different incision sites of phacoemulsification on trabeculectomy bleb function: prospective case-control study

**DOI:** 10.1186/s12886-017-0500-9

**Published:** 2017-06-26

**Authors:** Mohamed Anbar, Hatem Ammar

**Affiliations:** 1 Sohag University, Faculty of Medicine, Sohag, Egypt; 2 Ophthalmology Department, Sohag University Hospital, Sohag, 82511 Egypt

**Keywords:** Trabeculectomy, Glaucoma, Phacoemulsification, Trabeculectomy bleb

## Abstract

**Background:**

The aim of this study was to compare superior and temporal clear corneal incisions of uneventful phacoemulsification and in-the-bag intraocular lens implantation on intraocular pressure control and the bleb morphology in eyes that have undergone previous successful trabeculectomy.

**Methods:**

In this Prospective case-control study**,** a total of 100 eyes of 100 patients previously undergone trabeculectomy without antimetabolites, divided into two groups. Group A (temporal group) including 50 patients underwent phacoemulsification with a temporal corneal incision and group B (superior group) including 50 patients underwent phacoemulsification with a superior corneal incision. Comparisons between the two groups were performed after one year of follow-up regarding Intraocular pressure changes, bleb morphology score using the Wuerzburg bleb classification score and any added glaucoma medications.

**Results:**

At the last visit, the mean intraocular pressure for the temporal group was 17.55 ± 1.47 (*p* = 0.51) and for the superior group was 16.90 ± 1.71 (*p* = 0.85); the difference between the two groups was insignificant (*p* = 0.21). Regarding the bleb morphology, the mean bleb morphology score in the temporal group was 10.50 ± 0.95 (*p* = 0.19) and for the superior group was 10.20 ± 1.06 (*p* = 0.01).There was an insignificant difference in the bleb grading morphology regarding both groups (*p* = 0.35).

**Conclusion:**

Our study demonstrates that phacoemulsification whether done with a clear temporal or clear superior wound, does not affect intraocular pressure, bleb morphology or function after one year of follow-up in eyes following previous successful trabeculectomy. Furthermore, cataract surgery may be performed safely in eyes with functioning filtering blebs.

**Trial registration:**

ISRCTN91835217 ‘retrospectively registered’ Date Of registration 6/6/2017

## Background

It has been confirmed that filtering surgery for glaucoma is an accelerating factor for cataract formation and that cataract extraction becomes pivotal in the majority of eyes that undergo trabeculectomy [1].

There have many studies reporting the long-term intraocular pressure control (IOP) after cataract surgery in eyes with functioning filtering blebs, mainly from the period of intracapsular or extracapsular cataract extraction. It has been reported that cataract extraction using the extracapsular technique leads to a somewhat reduction in the bleb function [2–5]. The effect of phacoemulsification on intraocular pressure control in eyes that have undergone trabeculectomy seems to vary. Some studies have shown that uneventful phacoemulsification does not have any impact or influence the function of a good trabeculectomy blebs [6–8]. While others have confirmed worsening of the intraocular pressure and/or increase in the number of glaucoma medications after uncomplicated phacoemulsification [9–15]. However, it is important to differentiate between trabeculectomy bleb failure after cataract extraction from that which would occur due to the natural history of a filtration bleb function which fails over time.

The aim of this study was to compare superior and temporal clear corneal incisions of uneventful phacoemulsification and in-the-bag intraocular lens (IOL) implantation on IOP control and bleb morphology in eyes that had undergone previous successful trabeculectomy.

## Methods

The study was approved by the University Ethics Committee and followed the tenets of the Declaration of Helsinki. Participants were enrolled in a prospective, randomized control trial. This study was performed at the Department of Ophthalmology, Sohag University, Egypt. We obtained written informed consent from all participants.

All patients in this study had a previous successful trabeculectomy not augmented by antimetabolites and had well-controlled IOP, a well-functioning bleb, no preoperative glaucoma medications and a visually significant cataract before phacoemulsification.

A total of 100 eyes of 100 patients previously undergone trabeculectomy without antimetabolites divided into two groups. Group A (temporal group) including 50 patients underwent phacoemulsification with a temporal corneal incision and group B (superior group) including 50 patients underwent phacoemulsification with a superior corneal incision. All 100 patients underwent cataract surgery at least six months after the previous successful trabeculectomy surgery. All patients were assigned consecutively in both groups.

Ophthalmological examinations consisted of the best corrected visual acuity, slit lamp examination to detect the type of cataract and to make grading of the filtering bleb using the Wuerzburg bleb classification score [16], filtering bleb photography and Goldman applanation tonometry. At least three consecutive measurements were taken and the average of these measurements was taken as the preoperative IOP.

The following bleb morphological criteria were evaluated (Table 1): vascularization, corkscrew vessels, microcysts and encapsulation. A high bleb score (12 optimum) refers to a good function of the bleb.

**Table 1:** Wurzburg bleb classification score. Four parameters are evaluated including vascularity, crockscrew vessels, encapsulation and bleb microcysts

Parameters	Scoring
Vascularity	3 = Avascular 2 = similar to adjacent conjunctiva 1 = increased 0 = massive
Corkscrew vessels	3 = None 2 = in one third 1 = in two thirds 0 = entire bleb
Encapsulation	3 = None 2 = in one third 1 = in two thirds 0 = entire bleb
Bleb microcysts	3 = entire bleb 2 = lateral or medial of the flap 1 = over the scleral flap 0 = none

Patients were examined at day one, one week, one month, three months, six months, and one year postoperatively.Three basic parameters were selected as outcome measures and evaluated at each visit: IOP, bleb morphology and number of glaucoma medications added to further control of IOP. The exclusion criteria are patients with primary angle closure glaucoma, secondary glaucoma, any associated eye diseases, preoperative antimetabolite use and posterior capsule rupture and/or vitreous loss during phacoemulsification. Trabeculectomy failure was defined as an IOP above 21 mmHg at two consecutive visits during the follow-up period.

The postoperative inflammation was evaluated in all patients of both groups using slit lamp beam ±3 mm length and 1 mm width in the first hours postoperatively and through the first week.

### Trabeculectomy technique

Conjunctival flaps were fornix-based and the scleral Flaps were triangular of at least half thickness at the superior quadrant without antimetabolites application.

### Phacoemulsification technique

Phacoemulsification surgery was performed through a sutureless 2, 2 mm superior or temporal clear corneal incision. The temporal incisions were placed 180° for the right eyes and in 0° in the left eyes, while the superior incisions were placed 5 h clock temporal to the bleb. At 95° in the right eyes and at 85° in the left eyes. The Alcon Infinity phacoemulsification machine was used in all cases (Alcon Fort Worth, TX, USA). The stop-and chop technique was the preferred technique for nucleus emulsification. A one-piece hydrophobic acrylic foldable intraocular lens (IOL) (Alcon AcrySof SN60) was implanted into the capsular bag in all eyes. Meticulous removal of all viscoelastic at the end of surgery was undertaken.

### Statistical analysis

Data were analyzed using STATA intercooled version 12.1. Quantitative data are represented as the mean and standard deviation. Data were analyzed using Student’s t-test to compare the means of two groups and paired t-tests to compare data at different time intervals to the preoperative data. Qualitative data are presented as the number and percentage and compared using the chi-squared test. Graphs were produced using Excel or STATA.

## Results

The study consisted of 100 eyes had a significant cataract following trabeculectomy not augmented with antimetabolites. The characteristics of the studied population as well as the preoperative cataract morphology in both groups are shown in Table 2.

**Table 2:** Characteristics of the studied population

Characteristics	Temporal phaco *N* = 50	Superior phaco *N* = 50	*P* value
Age	57.40 ± 6.77	56.60 ± 6.30	0.58
Gender			0.75
Female	27 (55.00%)	30 (60.00%)	
Male	23 (45.00%)	20 (40.00%)	
Duration of Trabeculectomy	2.48 ± 1.02	2.73 ± 1.32	0.51
Cataract Morphology			
Nuclear Cataract Grad 2	13	10	
Grade 3	17	13	
Cortical	10	11	
Posterior subscapsular	6	8	
Mature (White)	4	8	

Characteristic of the studied population. The mean age, sex and the mean duration of trabeculectomy operation of both groups are mentioned as well as the Preoperative Cataracts Morphology in both groups

Most of the patients in our study had primary open-angle glaucoma (44/50 in temporal group and 40/50 in superior group). The mean period between trabeculectomy and phacoemulsification was 2.48 ± 1.02 years for the temporal group and 2.73 ± 1.32 for the superior group (*p* = 0.51). Both groups were clinically matched regarding age (*p* = 0.58) and gender (*p* = 0.75). Both groups were clinically and statistically matched regarding the preoperative IOP (*P* = 0.40) and the bleb morphology score (*p* = 0.64). Patients in both groups were followed up for one year postoperatively.

The mean IOP at each follow-up visit in both groups is shown in Table 3. Regarding the temporal group, in the first postoperative week, the IOP was higher by 1 or 2 mmHg in in 22 eyes (45%), lower in 3 eyes (5%) and unchanged in 25 eyes (50%). In the superior group, the IOP was higher by 1 mmHg in 25 eyes (50%) and unchanged in 25 eyes (50%).

**Table 3:** comparison between the temporal and superior groups regarding the mean intraocular pressure preoperatively and at each follow-up visit

Characteristics	Temporal phaco *N* = 50	Superior phaco *N* = 50	*P* value
Preoperative	17.40 ± 1.64	16.95 ± 1.73	0.40
Postoperative During the first week	17.80 ± 1.58	17.50 ± 1.40	0.53
Postoperative (1 month)	17.45 ± 1.43	17.35 ± 1.66	0.84
Postoperative (3 month)	17.40 ± 1.39	16.90 ± 1.62	0.30
Postoperative (6 month)	17.50 ± 1.36	16.85 ± 1.63	0.18
Postoperative (1 year)	17.55 ± 1.47	16.90 ± 1.71	0.21
P1 (Post op. (1 week) compared to pre op.)	0.03	0.01	
P2 (Post op. (1 month) compared to pre op.)	0.77	0.09	
P3 (Post op. (3 month) compared to pre op.)	1.00	0.84	
P4 (Post op. (6 month) compared to pre op.)	0.63	0.71	
P5 (Post op. 1 year) compared to pre op.)	0.51	0.85	

Throughout the follow up period the difference in preoperative intraocular pressure and postoperative pressure was insignificant and the intraocular pressure regarding both groups was also insignifica

At the last visit, the mean IOP in the temporal group was 17.55 ± 1.47 and for the superior group was 16.90 ± 1.71. The difference between the two groups was insignificant (*p* = 0.21). The difference between preoperative and postoperative IOP within the same group was found to be statistically insignificant throughout the follow up period (*p* = 0.51 in temporal group, *p* = 0.85 in the superior group).

### Bleb morphology

The preoperative bleb morphology score in both groups was statistically insignificant (*p* = 0.64). The mean preoperative bleb grading was 10.65 ± 0.99 for the temporal group and 10.50 ± 1.05 for the superior group.

After one year, the mean bleb morphology score in the temporal group was 10.50 ± 0.95 and for the superior group was 10.20 ± 1.06.There was an insignificant difference in the bleb grading morphology score regarding both groups (*p* = 0.35). During the first week after cataract surgery, there was a significant increase in bleb vascularity in both groups (*p* < 0.0001). Table 4 shows the comparison of the temporal and superior groups regarding pre- and postoperative bleb scores. The difference between preoperative and postoperative bleb scoring morphology within the same group was found to be statistically insignificant throughout the follow up period (*p* = 0.19 in temporal group, *p* = 0.01 in the superior group).

**Table 4:** Comparison of the temporal and superior groups regarding pre- and postoperative bleb scores

Characteristics	Temporal phaco *N* = 50	Superior phac *N* = 50	*P* value
Preoperative	10.65 ± 0.99	10.50 ± 1.05	0.64
Postoperative (1 week)	9.95 ± 1.00	9.90 ± 0.91	0.87
Postoperative (1 month)	10.35 ± 0.93	9.90 ± 0.91	0.13
Postoperative (3 month)	10.40 ± 0.88	10.20 ± 1.06	0.52
Postoperative (6 month)	10.45 ± 0.89	10.20 ± 1.06	0.42
Postoperative (1 year)	10.50 ± 0.95	10.20 ± 1.06	0.35
P1 (Post op. (1 week) compared to pre op.)	<0.0001	<0.0001	
P2 (Post op. (1 month) compared to pre op.)	0.03	<0.0001	
P3 (Post op. (3 month) compared to pre op.)	0.06	0.01	
P4 (Post op. (6 month) compared to pre op.)	0.10	0.01	
P5 (Post op. 1 year) compared to pre op.)	0.19	0.01	

Throughout the follow up period the difference in preoperative bleb score and postoperative score was insignificant and the bleb score regarding both groups was also insignificant

As regard the postoperative inflammation, in all cases the aqueous flare was ranged from complete absence to faint flare and the aqueous cells from no cells to rare cells with which disappeared completely in the first week postoperatively after starting postoperative treatments.

### Glaucoma medications

In terms of the number of glaucoma medications, there were five patients in the temporal group and seven patients in the superior group who required glaucoma medication six months after cataract surgery. These medications were added as neuroprotection since we found that the visual fields of these five patients were getting worse despite good control of IOP.

## Discussion

Several studies have previously assessed the effect of cataract extraction on IOP and bleb morphology [2, 9, 17, 18]. These studies investigated the effect of cataract removal by extracapsular cataract extraction (ECCE) or by phacoemulsification on IOP and bleb morphology in comparison with the normal course of trabeculectomized eyes without cataract extraction.

To the best of our knowledge, this is the first study to compare two different surgical sites for phacoemulsification and their effect on IOP and bleb morphology in patients with previous trabeculectomy.

In the present study, the mean IOP at the last visit was not significantly higher than that before cataract surgery in both study groups.

Park et al. [6] in a retrospective, case-control study, showed no adverse effect of temporal clear corneal phacoemulsification on IOP control in eyes with filtering blebs. In two retrospective studies done by Manoj et al. [2] and Mietz et al. [7], similar to the findings of Park et al., eyes having a well-controlled IOP after trabeculectomy tend to have a good prognosis after subsequent cataract surgery.

In a recent study done by longo et al. [19], they found that cataract surgery reduces the function of filtering bleb in some eyes. They mentioned some factors which may be associated with good outcomes as the previous use of mitomycin C during trabeculectomy, longer time from trabeculectomy to cataract surgery, and good preoperative aspect of the bleb.

Inal et al. [1] in a prospective study done on 30 patients who underwent clear corneal phacoemulsification after previous trabeculectomy, they found that uneventful clear corneal phacoemulsification was not associated with any worsening effect on IOP control in eyes with filtering blebs.

In another prospective study performed by Klink et al. [14], cataract extraction using clear cornea phacoemulsification in eyes with filtering blebs was associated with an elevation in IOP or with impaired filtering bleb morphology.

On the other hand, Casson et al. [4, 5] reported that a greater percentage of filtered eyes needed additional glaucoma medication following superior quadrant clear corneal phacoemulsification. Crichton & Kirker [10], Derbolav et al. [12] and Shingleton et al. [20] agreed with the previous study done by Casson et al.

In another prospective controlled study, Swamynathan et al. [21] found that temporal corneal phacoemulsification can affect long-term IOP control after trabeculectomy augmented by usingantimetabolites.

These results were the same as those of Rahat Husain et al. [15] who stated that cataract surgery after trabeculectomy increases the risk of trabeculectomy failure. However, these two studies should not be compared to our study because we did not use any antimetabolites during trabeculectomy.

**Fig. 1:**
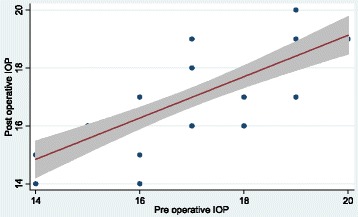
Scattergram of preoperative and postoperative IOP. Scattergram shows distribution of the mean preoperative IOP of this study is relatively high. This is because we did not use any antimetabolites during the antiglaucoma surgery

The mean preoperative IOP of this study is relatively high as shown in Fig. 1. This is because we did not use any antimetabolites during the antiglaucoma surgery.

It is worth mentioned that this relatively high preoperative IOP (17.17 ± 1.68) is the target IOP of the study cases. This relatively high preoperative IOP coincides with other old studies where antimetabolites were not also used as Ehrnrooth et al. [8](16.2 ± 4.9).

In another studies where antimetabolites were used, the mean preoperative IOP was lower. For example in longo et al. [19] and Swamynathan et al. [21] the preoperative IOP was 14.2 ± 2.35, 11.8+/−4.2 respectively. We have to mention that the target IOP was lower in these studies where antimetabolites were used so any postoperative changes will have a much bigger effect on IOP than the case in our study.

For the interpretation IOP regulation in filtered eyes, we have to consider the normal course of IOP after filtering surgery. A slight increase in IOP over time is common after trabeculectomy [22].

Since 1989, a number of observations and classifications of filtering blebs using morphological criteria have been described [16].

Several bleb grading systems were previously described In order to allow an accurate evaluation of bleb morphology and to predict early signs of failure. Some of these grading systems are the classification system described by Picht and Grehn [23, 24], the Indiana Bleb Appearance Grading Scale (IBAGS) [22] and the Moorefields Bleb Grading System

(MBGS) [25]. We used the Wuerzburg bleb classification score including four criteria (vascularization, corkscrew vessels microcysts and encapsulation) to evaluate bleb morphology [16].

In this study, bleb height was not included in the evaluation of bleb function and morphology because, in our opinion, none of the previous studies used a standardized method to evaluate bleb height.

Inal et al. [1] used their own classification system. They classified the bleb into “diffusely elevated” (microcystic and well-functioning); “moderately elevated” (moderately functioning) and “flat” (barely visible and non-functional. Klink et al. [14] used four criteria (vascularization, corkscrew vessels, microcysts and encapsulation) to establish a semi-quantitative score and calculated the bleb elevation separately using corneal thickness equivalents for quantification.

In our study, worsening of bleb morphology did not occur in either the control or study eye at the last visit. Previous studies have found that bleb appearance may change and the bleb function may decrease after phaco surgery, but severe fibrosis and total loss of bleb function have been rarely reported [1].

In a retrospective study done by Nguyen et al. [26] they found that phacoemulsification performed after trabeculectomy significantly increased rates of bleb failure in the following 12 months, but not at 24 months.

Manoj et al. [2] were unable to detect complete cicatrization of filtration blebs in any of their study eyes following phacoemulsification surgery.

Inal et al. [1] found worsening of bleb morphology (a decrease in bleb height and size) in both the control and study eyes, but after the one-year control visit bleb, morphology of the study and control eyes became similar.

They found gradual decrease in the success of filtering surgery and progressiveworsening of the IOP control in a time-dependent manner. Uneventful clear corneal phacoemulsification did not seem to have any additional deleterious influence on that course.

Klink et al. [14] found that 33%of filtering blebs were classified as “favorable” (>10 points) before phacoemulsification, and 27% remained “favorable” at the one-year visit. Hence, there was only a small general effect of phacoemulsification on bleb morphology as detectable with slit lamp biomicroscopy.

## Conclusion

Our study demonstrates that phacoemulsification, whether done with a clear temporal or a clear superior wound, did not affect the IOP or bleb morphology/function after one year of follow-up in eyes with previous successful trabeculectomy. Cataract surgery may be performed safely in eyes with functioning filtering blebs.

## Abbreviations

ECCE: Extracapsular cataract extraction
IOL: Intraocular lens
IOP: Intraocular pressure
Phaco: Phacoemulsification
POAG: Primary open-angle glaucoma
